# A Rare Case of Chronic Photophobia Associated With Oral Vancomycin Therapy: Exploring the Relationship

**DOI:** 10.7759/cureus.42145

**Published:** 2023-07-19

**Authors:** Serin Moideen Sheriff, Nonso Kingsley Chukwunyelu, Chinedu J Ezeafulukwe, Sandra S Kunnel, Omar A Hassan

**Affiliations:** 1 Department of Emergency Medicine, Lal Bahadur Shastri Hospital, Delhi, IND; 2 Department of Internal Medicine, Odesa National Medical University, Odessa, UKR; 3 Department of Internal Medicine, Sumy State University, Sumy, UKR; 4 Department of Internal Medicine, Windsor University School of Medicine, Cayon, KNA; 5 Department of Internal Medicine, Pushpagiri Institute of Medical Sciences and Research Centre, Thiruvalla, IND; 6 Department of General Medicine, Ondokuz Mayis University, Samsun, TUR

**Keywords:** adverse drug events, clostridium difficile colitis, chronic photophobia, oral vancomycin therapy, ocular symptoms

## Abstract

Vancomycin is a widely used tricyclic glycopeptide antibiotic for treating various Gram-positive infections, including *Clostridium difficile* colitis. Although considered generally safe, it has been associated with several side effects. In this case report, we highlight a rare adverse effect in which a patient experienced chronic photophobia following treatment with oral vancomycin. This sheds light upon a potential relationship between oral vancomycin therapy and photophobia, emphasizing the need for increased awareness in clinical practice and urging further investigation into this association.

## Introduction

*Clostridioides difficile* infection (CDI) occurs when the normal balance of gut bacteria is disrupted, allowing *Clostridioides difficile* spores to take hold. These spores can be transmitted through various means, such as fecal-oral transmission, exposure to contaminated surfaces, or as a result of contact with infected individuals [1].

In the year 2017, the Infectious Diseases Society of America and the Society for Healthcare Epidemiology of America issued updated guidelines to treat the initial episode of CDI [2]. These guidelines recommended two treatment options: a 10-day course of oral vancomycin at a dose of 125 mg orally four times daily or a 10-day course of oral fidaxomicin at 200 mg twice daily [2]. Based on pharmacokinetic data, oral administration of vancomycin has become the preferred method for managing *Clostridium difficile *colitis due to its poor absorption from the gastrointestinal tract [3,4]. Nevertheless, studies have demonstrated that even with a dose exceeding 500 mg for ≥ 10 days, there is an elevated risk of systemic absorption and subsequent detection of measurable levels in the bloodstream [5]. This case report documents the rarely reported instance of a patient experiencing chronic photophobia after oral vancomycin therapy. The unique nature of this case unveils a potential association between vancomycin treatment and the development of persistent photophobia.

## Case presentation

A female patient, aged 32, visited her primary care physician, reporting pain in both eyes and associated visual disturbances for the past six months. The pain experienced is dull in nature, retro-orbital in location, and unrelated to the movement of the eyeballs. It typically occurs during the daytime and diminishes at night. The patient observed that her pain intensified whenever she exposed herself to sunlight, whereas seeking refuge indoors with closed curtains and minimal lighting provided relief. According to the patient’s husband's observation, the patient has been deliberately avoiding watching TV in a dark room and prefers to keep the lights off while at home. Recently, the patient went on a road trip with her friends, when she gradually started experiencing bilateral eye pain accompanied by a brief period (five minutes) of blurry vision and fluttering of her upper eyelids. The visual blurring and fluttering quickly resolved after covering her eyes with a thick layer of blanket. During the medical history discussion, the patient mentioned that these symptoms began shortly after she completed a 10-day course of 125 mg oral vancomycin, taken four times daily, for *Clostridium difficile* colitis. Six months ago, the patient had been experiencing watery diarrhea and vomiting for nearly a month. Initially, she was misdiagnosed with acute gastroenteritis. However, when the symptoms relapsed, a stool ​polymerase chain reaction test was conducted, which came back positive for *Clostridium difficile* toxin. Notably, there were no signs of photophobia during this one month. Symptoms of photophobia emerged only after the patient began oral vancomycin therapy.

​The patient has no previous record of refractive errors, migraine, eye trauma, or sexually transmitted diseases. Additionally, the patient takes no regular medications and has no chronic medical conditions or significant family medical history. The patient's physical examination suggested fully conscious, oriented, and aware of time, place, and person. Her eyes appear normal with no external abnormalities. Visual acuity, pupil reaction, and extraocular muscle movements are within normal limits. There are no signs of conjunctival injection, discharge, or corneal abnormalities. The heart sounds were normal on auscultation, with no murmurs detected, and the bilateral lung fields were clear. Blood pressure was 110/70 mmHg, heart rate was 75 beats per minute, respiratory rate was 16 breaths per minute, temperature was 98.6°F, BMI was 22.4 kg/m2, and oxygen saturation in room air was 98%. The laboratory investigations, encompassing a complete blood count, comprehensive metabolic panel, estimated glomerular filtration rate, thyroid-stimulating hormone, glycated hemoglobin HbA1c, and lipid panel, all of which yielded normal results. Following the assessment, it was determined that the patient required additional ophthalmic investigation, leading to a referral to the department of ophthalmology.

The patient underwent a visual examination test (Table 1) at the ophthalmology outpatient department (OPD). The test results revealed normal distance and near vision, indicating that the visual acuity was within the normal range. There were no visual field defects, and the extraocular muscle function was normal. However, it was noted that the patient exhibited an abnormal symptom of photophobia, which refers to sensitivity to light. Furthermore, a color vision test (Table 2) was conducted, demonstrating a normal perception of color. The fundus examination (Table 3) revealed no abnormalities in crucial structures such as the optic nerve, blood vessels, and retina. Additionally, the intraocular pressure was found to be within the expected range. Despite the absence of any prior history of eye conditions, trauma, or neurological disorders, it remains crucial to conduct a comprehensive evaluation to determine the underlying cause of photophobia. In this particular case, drug-induced photophobia is considered a potential cause. For symptomatic relief, the patient was advised to use photochromatic glasses and avoid direct exposure to light which can help mitigate the discomfort associated with sensitivity to light.

**Table 1:** Visual examination report N: near

Test name	Left eye	Right eye
Visual acuity (distance)	6/6 (normal)	6/6 (normal)
Visual acuity (near)	N5 (normal)	N5 (normal)
Visual field	No apparent visual field defect (normal)	No apparent visual field defect (normal)
Muscle function	Normal	
Photophobia	Present (abnormal)	
External eye	Normal	

**Table 2:** Color vision report

Test name	Left eye	Right eye
Ishihara chart	Identifies all colors correctly (normal)	Identifies all colors correctly (normal)
Eldridge green lantern (small/medium/large apertures)	Identifies red, green, and yellow light correctly (normal)	Identifies red, green, and yellow light correctly (normal)

**Table 3:** Fundus examination report IOP: intra-ocular pressure, CD: cup to disc ratio

Components	Left eye	Right eye
Optic disc	Normal	Normal
Macula	Normal	Normal
Periphery	Normal	Normal
CD	0.3 (normal)	0.3 (normal)
IOP reading	14.0 (normal)	14.4 (normal)

## Discussion

CDI is one of the most common healthcare-associated infections. Apart from a rise in both the frequency and severity of infections, nearly a quarter of patients experience recurrent infections [6]. The current guidelines recommend oral vancomycin as the primary treatment for initial CDI due to its pharmacokinetic properties and limited absorption within the intestines [2-4]. However, conflicting clinical evidence exists concerning the extent of systemic absorption following oral administration [5]. A prospective observational study revealed detectable vancomycin levels in 68% of cases [5]. Risk factors associated with systemic drug exposure included ICU admission, therapy duration of 500 mg daily for ≥ 10 days, severe CDI presence, renal dysfunction, inflammatory GI conditions, and concurrent use of vancomycin retention enemas [5]. Previously, there have been reports of ocular hypersensitivity associated with the topical use of vancomycin in patients [7]. Additionally, there is existing literature about vancomycin-associated light sensitivity found in phase 4 clinical trials conducted on people who were taking vancomycin hydrochloride from the FDA [8]. This is possibly the first documented instance of photophobia in a female patient after undergoing oral vancomycin therapy. Therefore, healthcare providers should be aware of this potential side effect before prescribing the medication.

Considering that the patient's exposure to vancomycin occurred six months before the OPD visit, there is no necessity to check the blood levels of vancomycin. It can be inferred that the patient was possibly exposed to systemic absorption, subsequently resulting in a chronic adverse event. Based on the clinical evidence and findings presented in this case report, it is plausible to consider photophobia as a potential adverse effect of oral vancomycin therapy.

## Conclusions

This case report highlights a significant and potentially adverse effect of oral vancomycin therapy associated with chronic photophobia. The patient presented with ocular discomfort and sensitivity to light, which persisted even after discontinuing the medication for six months. Although the exact mechanism underlying this adverse effect remains unclear, it is crucial to give due consideration and conduct comprehensive studies regarding the potential for systemic absorption and the occurrence of chronic photophobia. Furthermore, additional research and pharmacovigilance efforts are needed to better understand the underlying pathophysiology of vancomycin-induced photophobia. This will facilitate the development of preventive strategies and alternative treatment options for patients who require vancomycin therapy but are at high risk for ocular adverse effects.
